# Tea and Porridge Syndrome: A Rare Cause of Severe Hyponatremia

**DOI:** 10.7759/cureus.90051

**Published:** 2025-08-14

**Authors:** Somarajan Anandan, Sajeesh Rajendran, Sisira S Rajan, Anandhu Suresh

**Affiliations:** 1 Neurology, St Joseph's Mission Hospital, Anchal, IND; 2 Neurology, Welcare Hospital, Kochi, IND

**Keywords:** hyponatremia, low solute intake hyponatremia, low urine osmolality, syndrome of inappropriate antidiuresis, syndrome of inappropriate antidiuretic hormone secretion, tea and porridge syndrome, tea and toast syndrome

## Abstract

Hyponatremia is a very common electrolyte imbalance in clinical practice and can result from diverse etiologies. Initial evaluation includes assessment of serum and urine osmolality, along with urinary sodium levels. Only a few causes of hyponatremia present with a urine osmolality of less than 100 mOsm/kg. We describe a case of severe hyponatremia with low urine osmolality in a hypertensive woman who presented with insomnia and vomiting. Due to persistent vomiting, she had been consuming a liquid, carbohydrate-only diet with low salt intake and tea, leading to low solute intake hyponatremia, similar to the "tea and toast" syndrome commonly reported in Western countries. In this case, it is more appropriately called "tea and porridge" syndrome.

## Introduction

Hyponatremia is the most common electrolyte imbalance in clinical practice and can be due to a number of diseases or commonly used drugs [1,2]. Hyponatremia occurs in many neurological, gastrointestinal, renal, and respiratory diseases. Serum sodium can be called a biochemical ESR (erythrocyte sedimentation rate) as it occurs in a variety of diseases, and hyponatremia may be the first clue to a rare disease [3,4]. Hyponatremia is a disorder of water balance, and total body sodium can be normal, reduced, or increased in hyponatremia. It can be acute (<48 hours) or chronic (>48 hours). It can be mild (130-135 mEq/L), moderate (125-130 mEq/L), or severe (<125 mEq/L) [5]. As kidney requires solutes to excrete water, low solute intake limits the body’s capacity to excrete water. Main solutes excreted in urine are urea and electrolytes. As urea is formed from ingested proteins, low protein intake limits the body’s capacity to excrete water, and a normal water intake can cause hyponatremia in persons with poor protein and salt intake [6]. Classical examples for low solute intake hyponatremia are tea and toast syndrome and beer potomania, which are characterized by intake of calories and free fluids without much protein. Here we describe a case of low solute intake hyponatremia akin to tea and toast syndrome - tea and porridge syndrome - in a patient with hypertension who followed a bland diet.

## Case presentation

**Figure 1:**
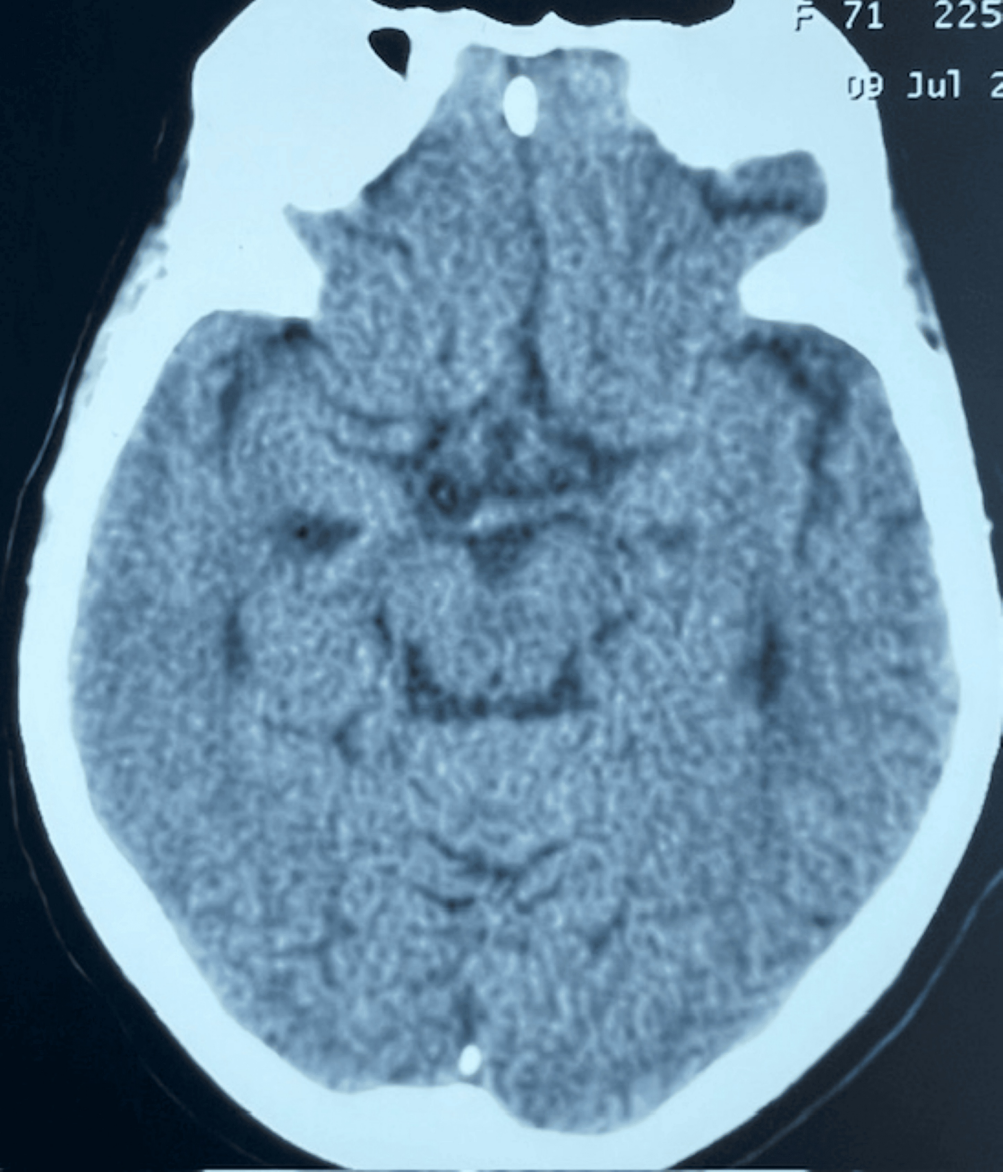
Computerised tomography of brain at the level of midbrain - normal The patient's age was entered incorrectly as 71 years by the external imaging center. Our medical records show her age as 68 years.

A 68-year-old female was admitted with a 5-day history of insomnia and vomiting. Relatives noticed mild swaying while walking for the last 2 days. There was no weakness or upper limb incoordination. There was no history of fever, headache, or altered sensorium or memory impairment. There was no incontinence or polyuria. Because of vomiting, she was taking only rice porridge without salt and black tea. There was no history of any excessive water drinking. There was no history of any beer or drug abuse. She had a history of severe aortic stenosis due to a bicuspid aortic valve and underwent bioprosthetic aortic valve replacement in 2018. She had been hypertensive since 4 years before presentation and was on telmisartan, cilnidipine, and hydrochlorothiazide at the time of admission. On examination, her blood pressure was 150/80 mmHg, and her pulse was 78 beats per minute. There were no signs of dehydration. Nervous system examination showed an alert and oriented woman with preserved recent memory. There was no weakness. She had tandem ataxia. In view of vomiting and ataxia, a computerized tomography of the brain was done, which was normal (Figure 1). Biochemical evaluation showed severe hyponatremia (109.8 mEq/L), hypouricemia (1.9 mg/dL), and low urinary sodium (14 mEq/L) and low urinary osmolality (71.9 mOsm/Kg), suggestive of a low solute intake state (Tables 1, 2).

**Table 1:** Blood parameters FENa: Fractional excretion of sodium, FEK: Fractional excretion of potassium, FEUA: Fractional excretion of uric acid, TSH: Thyroid-stimulating hormone, T4: Thyroxine

Parameter	Result	Normal range
Random blood sugar	96 mg/dL	80-140 mg/dL
Urea	22 mg/dL	20-45 mg/dL
Creatinine	0.59 mg/dL	0.6-1.2 mg/dL
Sodium	109.8 mEq/L	136-145 mEq/L
Potassium	3.6 mEq/L	3.5-5.5 mEq/L
Chloride	80 mEq/L	101-109 mEq/L
Bicarbonate	19 mmol/L	22-26 mmol/L
Uric acid	1.9 mg/dL	2.6-6 mg/dL
S. Cortisol (8 AM)	18.36 µg/dL	6.6-22.6 µg/dL
TSH	3.077 mIU/mL	0.35-5.5 mIU/mL
Free T4	1.1 ng/dL	0.6-1.1 ng/dL
FENa	0.8%	>0.5%
FEK	10.46%	4-16%
FEUA	13.54%	4-11%

**Table 2:** Urine biochemistry

Parameter	Result	Normal range
Urine osmolality	71.9 mOsm/Kg	50-1200 mOsm/Kg
Urine sodium (Na)	14 mEq/L	>20 mEq/L
Urine potassium	06 mEq/L	25-125 mEq/L
Urine chloride	15 mEq/L	110-250 mEq/day
Urine uric acid	4.1 mg/dL	250-750 mg/day
Urine creatinine	9.4 mg/dL	20-320 mg/dL
Urine pH	6.5	4.6-8

As the blood investigations showed profound hypo-osmolar hyponatremia (serum sodium (Na) 109.8 mEq/L, serum osmolality 234 mOsm/Kg), we sent blood and urine for a hyponatremia panel, which we send for all patients with moderate to severe hyponatremia. As she was on thiazides, it was stopped. She was treated with 3% saline, water restriction, and a high-protein diet. Her serum sodium improved to 116 mEq/L by the next day and to 126 mEq/L by the fourth hospital day, and she became asymptomatic. Even though telmisartan can cause hyponatremia with or without hyperkalemia, it was continued.

## Discussion

Syndrome of inappropriate antidiuretic hormone secretion (SIADH), better called syndrome of inappropriate antidiuresis (SIAD), is the most common cause of euvolemic hyponatremia [4]. It can be caused by a variety of diseases affecting different parts of the body and by a growing list of medications [7]. Urine osmolality >100 mOsm/Kg is an essential criterion, but urine sodium can be lower than the guidelines if salt intake is poor. It is also characterized by elevated fractional excretion of uric acid (FEUA>11%), which is often useful when urinary sodium cannot be relied upon, as in patients on frusemide. Another differential diagnosis is primary polydipsia, which will have characteristic excess fluid intake and can mimic tea and toast syndrome (Table 3). FEUA can be elevated in a few other conditions causing hyponatremia, and is a useful tool when urine sodium is borderline (20-50 mEq/L)(Table 4).

**Table 3:** SIAD vs. tea and porridge syndrome vs. primary polydipsia SIAD: syndrome of inappropriate antidiuresis, FEUA: fractional excretion of uric acid

Parameter	SIAD	Tea and porridge syndrome	Primary polydipsia
Serum Osmolality	Low	Low	Low
Volume status	Euvolemic	Euvolemic	Euvolemic
Serum uric acid	Low or normal	Normal or low	Low or normal
Urine osmolality	>100 mOsm/kg	<100 mOsm/kg	<100 mOsm/kg
Urine Na	>40 mEq/L	<20 mEq/L	Often <20 mEq/L
FEUA	High	High	Normal or high

**Table 4:** Hyponatremia with elevated FEUA and Hyponatremia with low urine osmolality SIAD: Syndrome of inappropriate antidiuresis, FEUA: Fractional excretion of uric acid

Hyponatremia with elevated FEUA	Hyponatremia with low urine osmolality
SIAD	Primary polydipsia
Cerebral/Renal salt wasting syndrome	Beer potomania
Primary polydipsia	Tea and toast syndrome (low solute intake)
Beer potomania	During recovery from SIAD
Tea and toast syndrome (low solute intake)	Reset osmostat
Isolated cortisol deficiency	
Drugs: Losartan	

Thiazides are an FDA-approved class of medication to treat hypertension and are a very common cause of hyponatremia. Generally, it is characterized by increased urinary sodium with increased urinary osmolality. Thiazides can cause severe hyponatremia in hospitalized patients and are associated with high morbidity and mortality. Thiazide diuretics inhibit sodium chloride (NaCl) reabsorption in the distal convoluted tubule, the main diluting site of the nephron. Thiazide diuretics interfere with maximum dilution of urine because sodium excretion is increased along with diminished free-water clearance, predisposing to hyponatremia. Thiazides cause two types of hyponatremias: one is hypovolemic hyponatremia due to its diuretic effect, and the other is euvolemic hyponatremia mimicking SIAD. Even patients who have done well on chronic thiazide therapy may develop severe hyponatremia if water intake increases [8]. In our patient, even though she was on thiazide diuretics, low urinary sodium and very low urinary osmolality pointed towards a low solute intake hyponatremia. 

Our patient had hypo-osmolar euvolemic hyponatremia with urinary osmolality <100 mOsm/Kg. Differential diagnoses were primary polydipsia and low solute intake states like beer potomania, tea and toast syndrome (in Western countries, where bread is the staple diet) [9]. In low-income countries, where rice is the staple food, porridge without much protein or salt intake and excess water or tea can lead to hyponatremia (Tea and Porridge syndrome).

Hyponatremia with low urine osmolality is caused by only a few conditions, and urine osmolality determination is very important in diagnosis (Table 4). Urinary sodium is usually low in these conditions. Urinary sodium can below low in hypovolemic hyponatremia, hypervolemic hyponatremia, and salt-depleted SIAD, but urine osmolality will be >100 mOsm/Kg, underpinning the importance of urinary osmolality in the work-up of hyponatremia [10]. A cost-effective hyponatremia panel that picks up the most common causes of hyponatremia is suggested in Table 5.

**Table 5:** Hyponatremia panel TSH: Thyroid-stimulating hormone, T4: thyroxine

Blood	Urine
Blood sugar	Osmolality
Urea	Sodium
Creatinine	Potassium
Sodium	Chloride
Potassium	Creatinine
Chloride	Uric acid
Uric acid	
S. Osmolality	
TSH	
Free T4	
Cortisol	

## Conclusions

Hyponatremia is a very common electrolyte abnormality in clinical practice and can be asymptomatic or can have severe manifestations, including altered sensorium, seizures, or coma. Finding out the etiology is very important for proper management and to prevent recurrence. In this era of panel-based investigations, a hyponatremia panel is a cost-effective way to classify and clarify the etiology of hyponatremia.

Even though hyponatremia is a common side effect of thiazide diuretics, not all hyponatremia in patients on thiazide diuretics may be due to it. Here we describe a rare cause for euvolemic hypotonic hyponatremia due to low solute intake - tea and porridge syndrome - in a female with hypertension on a thiazide-based anti-hypertensive regimen. This case highlights the importance of testing urine osmolality and urine sodium in all cases of hyponatremia, even if there is an obvious cause, like a thiazide diuretic.
